# sST2 and Heart Failure—Clinical Utility and Prognosis

**DOI:** 10.3390/jcm12093136

**Published:** 2023-04-26

**Authors:** Magdalena Dudek, Marta Kałużna-Oleksy, Jacek Migaj, Filip Sawczak, Helena Krysztofiak, Maciej Lesiak, Ewa Straburzyńska-Migaj

**Affiliations:** 1 1st Department of Cardiology, Poznań University of Medical Sciences, 61-848 Poznań, Poland; 2 Heliodor Swiecicki Clinical Hospital in Poznan, 60-355 Poznań, Poland; 3 Department of Cardiology, University Hospital in Opole, 45-401 Opole, Poland

**Keywords:** sST2, heart failure, biomarker, HFrEF

## Abstract

New parameters and markers are constantly being sought to help better assess patients with heart failure (HF). ST2 protein has gained interest as a potential biomarker in cardiovascular disease. It is known that the IL-33/ST2L system belongs to the cardioprotective pathway, which prevents the fibrosis, hypertrophy, and apoptosis of cardiomyocytes and also inhibits the inflammatory response. Soluble ST2 (sST2) is involved in the immune response and secreted in response to the mechanical overload of the myocardium, thus providing information on the processes of myocardial remodeling and fibrosis. A total of 110 hospitalized patients diagnosed with heart failure with reduced ejection fraction (HFrEF) were included in the study. Clinical and biochemical parameters were studied. During the follow-up, 30.9% patients died and 57.3% patients reached the composite endpoint. Using ROC curves, the reference cut-off point for sST2 was determined to be 45.818 pg/mL for all-cause deaths. Significantly higher concentrations of inflammatory parameters and natriuretic peptides were found in the group of patients with higher sST2 concentrations. sST2 protein is an independent risk factor for all-cause deaths of patients with HFrEF.

## 1. Introduction

Chronic heart failure (CHF) is a serious clinical syndrome and a cause of medical and social problems in industrialized countries [1]. HF is one of the major global health problems. It is estimated that in developed countries, HF affects up to 2% of the adult population [2]. A continuous increase in the prevalence of HF has been suggested, while the current prevalence is estimated to be approximately 26 million patients [3]. It should be noted that CHF significantly reduces patients’ quality of life, is an important cause of hospitalization, and the 5-year mortality rate is higher than that caused by many common malignancies [4,5,6]. Despite significant progress in understanding the mechanisms of HF development, the implementation of prophylaxis, and the introduction of new therapeutic methods, the prognosis of this group of patients is still poor. New parameters and markers are constantly being sought to help better assess patients with HF, which could translate into better care and an improved prognosis.

ST2 (suppression of tumorigenicity 2) protein has gained interest as a potential biomarker in cardiovascular disease. It is known that the IL-33/ST2L system belongs to the cardioprotective pathway, which prevents the fibrosis, hypertrophy, and apoptosis of cardiomyocytes and also inhibits the inflammatory response. ST2 is involved in the immune response and is secreted in response to the mechanical overload of the myocardium, thus providing information on the processes of remodeling and fibrosis [7]. Soluble ST2 (sST2) acts as a decoy receptor directly bound to IL-33 and reverses the beneficial effects of the IL-33/ST2 system [8]. Plasma levels of sST2 in the general population have been found to be associated with systolic blood pressure (SBP) values [8]. Patients with HF with preserved ejection fraction and hypertension had higher plasma levels of sST2 compared to patients without left ventricular hypertrophy. 

In recent years, studies on the use of sST2 protein as a biomarker in acute and chronic HF have been published. In recent years, studies have been published on the usefulness of the sST2 as a biomarker in acute [9] and chronic decompensated HF [10].

We aimed to assess the influence of sST2 concentration on the prognosis of patients with chronic HF.

## 2. Materials and Methods

### 2.1. Study Population

This study included patients with heart failure with reduced ejection fraction (HFrEF) who were hospitalized for the evaluation of CHF in the 1st Department of Cardiology at Poznan University of Medical Sciences in 2016–2017. The inclusion criteria were: having a left ventricle ejection fraction (LVEF) ≤45%, age ≥ 18 years, a stable period of illness (no hospitalization or need to administer intravenous diuretics due to exacerbation/decompensation of HF in the last 4 weeks), having undergone optimal pharmacological treatment according to the European Society of Cardiology 2016 Guidelines for Heart Failure [11], and having signed the informed consent form.

The study was conducted according to the guidelines of the Declaration of Helsinki, and the Ethics Committee of Poznan University of Medical Sciences approved it (No. 378/19). 

### 2.2. Analyzed Parameters

Clinical findings were collected, including age, gender, comorbidities, prescribed medications, HF etiology, New York Heart Association (NYHA) functional class, echocardiography assessment results, and cardiopulmonary exercise test results (CPET). We analyzed complete blood count and other laboratory parameters: sST2, N-terminal pro B-type natriuretic peptide (NT-proBNP), creatinine, aspartate aminotransferase (AST), alanine aminotransferase (ALT), electrolytes (sodium and potassium), creatinine, and high-sensitivity C-reactive protein (hsCRP). sST2 concentrations from peripheral blood were calculated via the ELISA method using The Presage^®^ ST2 Assay (Critical Diagnostics, San Diego, CA, USA) [12].

The left ventricular ejection fraction was calculated using echocardiographic Simpson’s method in accordance with the ESC guidelines [13]. Breathing gas analysis was performed using the Vmax29 Sensor Medics measurement module. Patients performed a symptom-limited maximal exercise test on a treadmill according to the RAMP protocol (the load increment when inclination and treadmill travel parameters were changed was 0.5 MET/min) or according to the Bruce protocol modified for HF. Follow-up data were obtained from electronic medical records or phone conversations with patients or their family members. If follow-up data were not obtainable using either previously mentioned methods, we used National Health Fund registry data. All patients were followed up at the outpatient clinic at 3 months, and then every 6 months after enrollment in the study. During the follow-up evaluation, the primary endpoint (all-cause death) was checked.

### 2.3. Statistical Analysis

Statistical analysis was performed using the STATISTICA 13 program by StatSoft, owned by Tibco Software Inc. (Palo Alto, CA, USA) Continuous variables are presented as mean ± standard deviation (SD) or median (lowest quartile–highest quartile), depending on the presence of normal distribution. Categorical variables are presented as numbers (%). 

Subjects were divided into two groups based on their survival. In addition, the optimal cut-off point for sST2 protein concentration to predict death from any cause was determined by analysis of the ROC curve, and participants were also grouped based on this cut-off point. Analysis of the time to death in the study groups was carried out using Kaplan–Meier analysis. The statistical significance threshold for all other tests was set at *p* < 0.05. 

## 3. Results

### Study Population

We enrolled 110 patients; 90% were men. The mean age was 53.1 ± 11.4 years, and the mean BMI was 28.2 ± 4.5 kg/m^2^. The most common comorbidities were arterial hypertension (44.6%), diabetes mellitus (25.5%), chronic obstructive pulmonary disease (12.7%), and atrial fibrillation (16.4%). The median LVEF was 23.3 ± 7.9%. In the study population, 54 patients presented with NYHA classes III and IV (49%), and 56 patients presented with NYHA classes I and II (50.8%). At the time of inclusion in the study, most of the patients were given an optimal treatment for HFrEF. The baseline characteristics are presented in Table 1.

**Figure 1:**
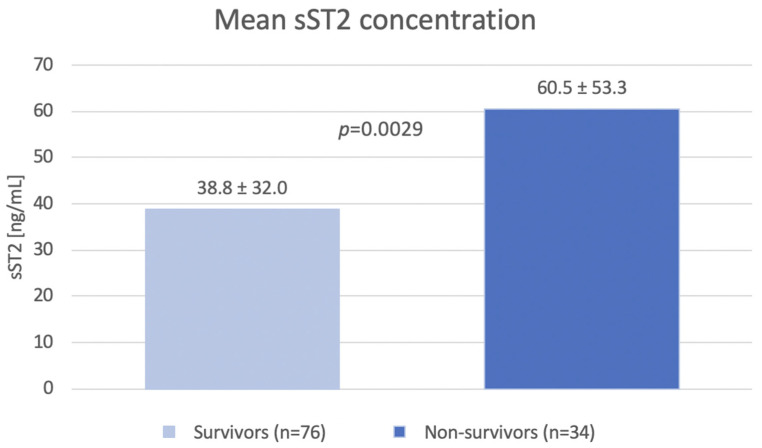
The mean values of sST2.

**Figure 2:**
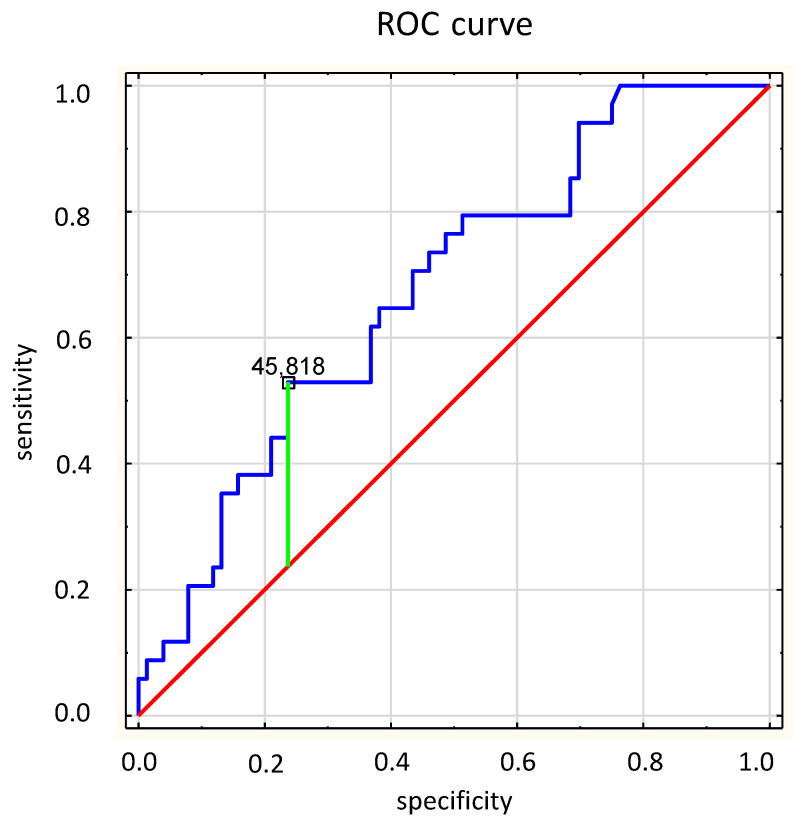
The reference cut-off point for sST2 for all-cause death.

sST2 concentration was measured in all patients. The mean value of sST2 was 45.5 ± 40.8 ng/mL. The SST2 level was significantly lower in the group of the survivors compared to that of the group of non-survivors (38.8 ± 32.0 ng/mL vs. 60.5 ± 53.3 ng/mL; *p* = 0.0029). During median follow up (2.7 years), 34 (30.9%) patients died and 63 (57.3%) patients achieved composite endpoint (all-cause death, hospitalization or intravenous diuretics requirement, or heart transplantation). During the follow-up period, 50 patients (45.5%) required hospitalization or intravenous diuretics. SST2 levels were significantly lower in the group of survivors as compared to the group of non-survivors (38.8 ± 32.0 ng/mL vs. 60.5 ± 53.3 ng/mL; *p* = 0.0029) (Figure 1). Based on collected data, the ROC curve was calculated for all-cause death, and the reference cut-off point for sST2 was determined to be 45.818 pg/mL (AUC 0.676, *p* = 0.0009) (Figure 2). According to the cut-off point, the study group was divided into two groups according to the sST2 values.

There were significant differences in the inflammatory parameters between groups. Patients with an sST2 concentration >45.8 pg/mL had significantly higher WBC (8.3 ± 2.0 vs. 7.3 ± 1.9; *p* = 0.0020), neutrophils (5.6 ± 1.5 vs. 4.6 ± 1.5, *p* = 0.0005), monocytes (0.6 ± 0.3 vs. 0.5 ± 0.1, *p* = 0.0016), and CRP values (8.2 ± 12.4 vs. 2.6 ± 3.1, *p* < 0.001) compared to those of the people with a lower concentrations. In addition, in this group the sodium concentration was significantly lower (137.9 ± 3.2 vs. 140.0 ± 2.1; *p* = 0.0005). The study groups differed in liver function parameters. AST and GGTP levels were significantly higher in patients with higher concentration of sST2. Significantly higher levels of BNP and NT-proBNP were observed in patients with higher levels of sST2. For BNP, the levels were 740.8 ± 779.5 pg/mL vs. 286.7 ± 318.9 pg/mL (*p* < 0.001), respectively, and for NT-proBNP, the levels were 3862.9 ± 5909.3 pg/mL vs. 1477.0 ± 1702.8 pg/mL (*p* < 0.001), respectively.

The LVEFs were similar in both groups (Table 2). In data obtained from cardiopulmonary exercise testing, we noticed significantly lower peak VO_2_ values (1.3 ± 0.3 L/min vs. 1.6 ± 0.6 L/min; *p* = 0.0030) in the subgroup of patients with higher sST2 protein values in comparison with those of the subgroup of people with sST2 ≤ 45.8 pg/mL. There were also significant differences in the VE/VCO_2_ slope, which was higher in patients with higher sST2 levels (35.7 ± 8.1 vs. 31.6 ± 6.9; *p* = 0.0188), and PETCO_2_, which was lower in patients with higher sST2 levels. 

**Figure 3:**
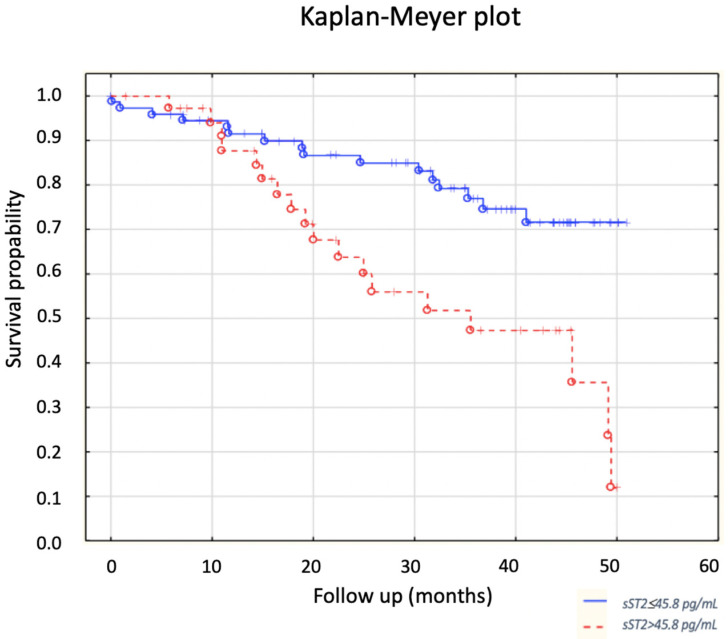
Influence of sST2 concentration on survival.

The probability of the primary endpoint depending on sST2 concentration was estimated using Kaplan–Meier curves (Figure 3). The Kaplan–Meier plot graphically shows that the probability of survival was significantly higher in the group of patients with low sST2 concentrations as compared to that of the group with high sST2 values (*p* = 0.0027).

In order to assess the influence of covariates on the relationship between sST2 concentration and the occurrence of the endpoint, multivariate analysis was performed. Based on the obtained and predicted relationships between dependent and independent variables, the following parameters were tested using multivariate models: systolic blood pressure, sST2, RDW, CRP, NT-proBNP, albumin, etiology of HF, peak oxygen uptake VE/VCO_2_ slope, and PETCO_2_.

Based on a multivariate regression model, it was shown that the independent predictors of death in stable patients with HFrEF were: peak oxygen uptake expressed in ml/kg/min (hazard ratio, 0.89; *p* = 0.0308), red blood cell distribution width (RDW) (hazard ratio, 1.10; *p* = 0.0074), and the concentration of sST2 (hazard ratio, 1.00; *p* = 0.0206).

## 4. Discussion

The most important finding of the presented analysis is that an increased concentration of sST2 is an independent risk factor for all-cause death in a group of patients with stable HFrEF.

Cardiovascular diseases, including HF, are the leading cause of morbidity and mortality in the industrialized world [14]. Despite significant progress in the understanding of pathophysiology, the introduction of prophylaxis, and modern therapeutic methods, HF is still a significant health and social problem which leads to a deterioration in the quality of life of patients and increased mortality. New markers are constantly being researched to help in the diagnosis and monitoring of patients with HF, as well as to help in identifying the group with the highest risk level. sST2 protein is gaining attention as a potential tool for monitoring the treatment of CHF and as a prognostic marker in this group due to its involvement in inflammatory processes and mechanical overload, as well as remodeling and fibrosis [12]. It was noted that sST2 concentrations in CHF patients were generally higher than they were in the healthy population [15].

In recent years, there has been an increased interest in this biomarker. Initially, sST2 was used in the assessment of HF decompensation, and also recently, for patients with CHF.

In the course of the PRIDE study (Pro-Brain Natriuretic Peptide Investigation of Dyspnea in the Emergency Department) [15], sST2 levels were measured in almost six hundred patients who were admitted to the emergency department due to dyspnea with suspected HF. It should be emphasized that the study group included both people with and without HF. The study [15] showed a significantly higher concentration of sST2 in the group of patients with dyspnea due to HF and the relationship between the concentration of sST2 and mortality in both groups of patients (higher concentrations of the biomarker suggest a higher risk of death). The publication by Mueller et al. [16] presents similar results: increased sST2 concentrations at the initial presentation in patients with acute heart failure (AHF) indicate an increased risk of death in the future; however, no cut-off value was reported for prognostic significance.

The presented study showed that the concentration of sST2 is an independent predictor of all-cause death in the group of patients with stable HFrEF. In patients who died during the observation, a significantly higher concentration of sST2 protein was found at the point of enrollment in the study (60.5 ± 53.3 pg/mL vs. 38.8 ± 32.0 pg/mL; *p* = 0.0029). Higher baseline sST2 levels are a strong predictor of death; 50% of patients with sST2 > 45.8 pg/mL died, while 21.6% patients from the group with lower levels died (*p* = 0.0025). Our study is one of the few studies that assessed the prognostic value of sST2 in patients with HFrEF, and the endpoint was assessed as death due to all causes.

Additionally, in a meta-analysis [17] conducted on a group of over 4000 patients, it was shown that the concentration of sST2 is an independent predictor of death from general and cardiovascular causes and an independent risk factor for HF-related hospitalization. Several fundamental differences between the Emdin study [17] and the one presented here should be emphasized. The study group [17] consisted of patients with HFrEF and heart failure with preserved ejection fraction (HFpEF) and with a higher mean age than that in our own study (68 years vs. 53 years). The follow-up was comparable in length to that in our own study (average 2.4 years), but the cut-off value for the sST2 level as a risk factor for death from all causes, death from cardiovascular causes, or hospitalization due to HF was 28 ng/mL. This is lower than it is in the analysis performed here, but the endpoints were different. It should be noted that research on sST2 is ongoing, and no specific cut-off value has been established for it as a prognostic indicator. Soluble ST2, together with galectin 3, is a parameter indicated by American experts as a promising marker that is helpful for predicting hospitalization and death in patients with HF, which may have even greater prognostic value than natriuretic peptides do. However, the 2017 update of the American College of Cardiology/American Heart Association HF guidelines [18] did not specify a cut-off value for sST2 for predicting the occurrence of death or adverse cardiovascular events. It seems that the purpose of including sST2 and galectin 3 in the guidelines [18,19] was to highlight the potential utility of these markers and to encourage further research in this area.

The long-term follow-up performed by Emdin et al. [17] assessed three endpoints of death from all causes, death from cardiovascular causes, and hospitalization due to HF. Cardiovascular deaths were not analyzed in our study as these data were not available. During the entire follow-up (mean 2.45 years) in this analysis, 34 patients (30.9%) died. This mortality rate is comparable to those in the analysis [17], where the all-cause mortality was 31% and the cardiovascular mortality was 22%. In the analyzed study, the inclusion criterion was a stable clinical condition at least 4 weeks before inclusion in the study. Despite the relatively good initial clinical condition of patients (mean NYHA 2.4), the applied optimal treatment, and the vast majority (82%) of patients having implanted cardioverter-defibrillators, mortality was not low. Another observation shows that the prognosis of patients is still not good, despite the use of an appropriate treatment. It seems that in patients with HF who are in a stable clinical condition, stabilization is only apparent.

Patients with higher sST2 concentrations had a higher percentage of diagnosed atrial fibrillation (*p* = 0.0039). The pathophysiologically increased hemodynamic load causes atrial distension, which is a well-known cause of the development of AF. The same mechanism can also stimulate sST2 secretion. A study by Chen et al. [20] showed that people with AF have a significantly higher concentration of soluble ST2 proteins than people with a sinus rhythm do. Higher sST2 concentrations in patients with AF are probably due to higher atrial pressures and higher heart rates than those in patients with a sinus rhythm [20].

Surprisingly, despite significantly higher concentrations of natriuretic peptides in non-surviving patients than the concentrations of those who survived, natriuretic peptides were not found to be an independent risk factor for all-cause mortality. They are recognized risk factors used in the diagnosis of HF [1] and prognosis of these patients [21,22,23,24]. Although significant correlations were observed between the concentrations of natriuretic peptides and the concentrations of sST2, people with higher sST2 concentrations have higher BNP (*p* < 0.001) and NT-proBNP (*p* = 0.001) values.

In patients with higher concentrations of sST2, we found higher levels of inflammatory markers. The available literature has shown that high concentrations of CRP protein and other markers have significant prognostic and therapeutic implications in HF [25]. In a study by Dupuy et al. [26] assessing sST2 and CRP levels in patients with CHF (*n* = 178), a combined multi-marker model including sST2 and CRP was presented. This study showed that the combination of high CRP (>6.4 mg/L) and elevated sST2 (>47.6 ng/mL) dramatically increased the risk of mortality, and a further association with NT-proBNP provided no additional information [26]. The cut-off point for sST2 in the study [26] was similar to the values determined in this analysis. The publication [26] confirmed that high sST2 values in combination with an elevated CRP are associated with an increased risk of all-cause or cardiovascular mortality, which is consistent with the results of this analysis.

Another parameter that is a well-established marker of poor prognosis for patients with HF is hyponatremia (defined as serum sodium concentration <135 mEq/L) [27,28,29,30]. In the study analyzed here, significant differences in sodium concentrations between the groups were observed. Significantly lower sodium concentrations were measured in subjects with higher sST2 values (137.9 ± 3.2 vs. 140.0 ± 2.1; *p* = 0.0005). The results obtained in the current analysis are consistent with those in available publications, which have proven that low sodium concentrations are associated with a higher mortality rate and are a predictor of hospitalization of patients with HF. No analyses on the correlation between sodium and sST2 concentrations were found in the available literature.

Our study had some limitations. According to ESC 2021 guidelines, sodium-glucose co-transporter 2 (SGLT2) inhibitors added to a therapy with ACE-I/ARNI/beta-blocker/MRA reduced the risk of CV death and worsening HF in patients with HFrEF. Our patients were involved in the study before these guidelines were published. Second, we analyzed a small number of patients in a single-center study. The majority of our study group were men; therefore, the cut-off point for sST2 in the group of women with HFrEF should be interpreted with caution.

## 5. Conclusions

sST2 protein concentration is an independent risk factor for all-cause death in patients with stable HF with reduced left ventricular ejection fraction. We suppose that sST2 may help to distinguish the group of patients with HFrEF who are at the highest risk, which will allow the better optimization of treatment and intensification of medical care in the form of more frequent check-ups of these patients.

## Figures and Tables

**Table 1:** Baseline characteristics of the studied group.

Parameter	Value (*n* = 110)
Age (years)	53.1 ± 11.4
Men	99 (90%)
BMI (kg/m^2^)	28.2 ± 4.5
SBP (mmHg)	108.0 ± 15.0
DBP (mmHg)	70.0 ± 9.0
LVEF (%)	23.3 ± 7.9
Duration of HF (months)	95.0 ± 86.0
Etiology of HF—CAD	56 (51%)
Etiology of HF—DCM	51 (46%)
NYHA class	2.4 ± 0.6
ICD	61 (56%)
CRT-D	29 (26%)
**Comorbidities**	
AF	18 (16.4%)
Arterial hypertension	49 (44.6%)
Diabetes mellitus type II	28 (25.5%)
Thyroid disease	21 (19.1%)
COPD	14 (12.7%)
Smoking at admission	14 (12.7%)
Smoking history	60 (54.6%)
**Biochemical parameters**	
sST2 (ng/mL)	45.5 ± 40.8
WBC (10^9^/L)	7.6 ± 2.0
RBC (10^12^/L)	4.8 ± 0.5
HGB (mmol/L)	8.9 ± 0.8
RDW (%)	15.2 ± 4.2
PLT (10^9^/L)	201.9 ± 52.8
ESR (mm/h)	9.5 ± 10.2
CRP (mg/L)	4.5 ± 7.9
Na^+^ (mmol/L)	139.3 ± 2.7
K^+^ (mmol/L)	4.4 ± 2.0
Creatinine (umol/L)	103.0 ± 24.1
Fasting glucose (mmol/L)	6.5 ± 2.4
NT-proBNP (pg/mL)	2257.9 ± 3797.3
TSH (uIU/mL)	2.0 ± 1.7
**CPET parameters**	
Peak VO_2_ (ml/kg/min)	17.4 ± 5.2
Peak VO_2_ (L/min)	1.5 ± 0.5
Peak VO_2_ (%)	54.3 ± 16.1
VE/VCO_2_ slope	33 ± 7.5
PETCO_2_ (mmHg)	33 ± 6.2
**Medications**	
Beta-blocker	108 (98.2%)
ACE-I/ARB	91 (82.7%)
ARNI	11 (10%)
MRA	96 (87.3%)
Loop diuretic	99 (90%)

BMI—body mass index; SBP—systolic blood pressure; DBP—diastolic blood pressure; NYHA—New York Heart Association; HF—heart failure; CAD—coronary artery disease; DCM—dilated cardiomyopathy; ICD—implantable cardioverter-defibrillator; CRT-D—cardiac resynchronization therapy defibrillator; AF—atrial fibrillation; COPD—chronic obstructive pulmonary disease; WBC—white blood cells; RBC—red blood cells; HGB—hemoglobin; RDW—red blood cell distribution width; PLT—platelets; ESR—erythrocyte sedimentation rate; CRP—C-reactive protein; Na^+^—sodium; K^+^—potassium; NT-proBNP—N-terminal prohormone of brain natriuretic peptide; TSH—thyrotropin; Peak VO_2_—peak oxygen consumption; VE/VCO_2_ slope—minute ventilation/carbon dioxide production; PETCO_2_—partial pressure of end-tidal carbon dioxide; LVEF—left ventricle ejection fraction; ACE-I—angiotensin-converting enzyme inhibitors; ARB—angiotensin II receptor blocker; ARNI—angiotensin receptor neprilysin inhibitor; MRA—mineralocorticoid receptor antagonist.

**Table 2:** Comparison of clinical and biochemical parameters between groups according to sST2 value.

Parameter	sST2 ≤ 45.8 (*n* = 74)	sST2 > 45.8 (*n* = 36)	*p*
Age (years)	53.0 ± 11.7	53.4 ± 11.0	0.7488
Men	89.20%	91.70%	0.6844
BMI (kg/m^2^)	28.9 ± 4.8	26.6 ± 3.7	**0.0146**
SBP (mmHg)	110.1 ± 16.3	104.9 ± 12.6	0.1808
DBP (mmHg)	72.2 ± 9.5	66.9 ± 8.1	**0.0090**
LVEF (%)	23.9 ± 8.0	21.9 ± 7.6	0.2595
Duration of HF (months)	101.5 ± 92.1	83.7 ± 72.7	0.5155
Etiology of HF—CAD	49%	56%	0.2950
Etiology of HF—DCM	50%	39%	0.2950
NYHA class	2.4 ± 0.6	2.6 ± 0.5	0.2164
ICD	50%	39%	0.2950
CRT-D	50%	39%	0.2950
**Comorbidities**			
AF	9.50%	30.60%	**0.0039**
Arterial hypertension	47.30%	38.90%	0.405
Diabetes mellitus type II	21.60%	33.30%	0.3411
Thyroid disease	17.60%	22.20%	0.56
COPD	12.20%	13.90%	0.7987
Smoking at admission	10.80%	16.70%	0.5877
Smoking history	54.10%	55.60%	0.5877
**Biochemical parameters**			
WBC (10^9^/L)	7.3 ± 1.9	8.3 ± 2.0	**0.002**
RBC (10^12^/L)	4.7 ± 0.4	4.8 ± 0.6	0.9368
HGB (mmol/L)	9.0 ± 0.7	8.9 ± 0.8	0.2094
RDW (%)	15.0 ± 4.9	15.8 ± 1.7	**0.0001**
PLT (10^9^/L)	197.8 ± 51.2	210.3 ± 55.7	0.2437
ESR (mm/h)	8.2 ± 7.3	12.4 ± 14.4	0.422
CRP (mg/L)	2.6 ± 3.1	8.2 ± 12.4	**<0.001**
Na^+^ (mmol/L	140.0 ± 2.1	137.9 ± 3.2	**0.0005**
K^+^ (mmol/L)	4.6 ± 2.4	4.2 ± 0.6	0.1958
Creatinine (umol/L)	101.2 ± 25.0	106.7 ± 21.9	0.2872
Fasting glucose (mmol/L)	6.5 ± 2.7	6.4 ± 1.7	0.5155
NT-proBNP (pg/mL)	1477.0 ± 1702.8	3862.9 ± 5909.3	**<0.001**
TSH (uIU/mL)	1.9 ± 1.7	2.0 ± 1.8	0.9671
**CPET parameters**			
Peak VO_2_ (mL/kg/min)	18.1 ± 5.5	15.8 ± 4.1	0.062
Peak VO_2_ (L/min)	1.6 ± 0.6	1.3 ± 0.3	**0.003**
Peak VO_2_ (%)	56.5 ± 16.0	49.5 ± 15.2	**0.0095**
VE/VCO_2_ slope	31.6 ± 6.9	35.7 ± 8.1	**0.0188**
PETCO_2_ (mmHg)	34.6 ± 5.8	29.7 ± 6.0	**<0.001**
**Medications**			
Beta-blocker	98.70%	97.20%	0.5993
ACE-I/ARB	82.40%	83.30%	0.9066
ARNI	12.20%	5.60%	0.2784
MRA	86.50%	88.90%	0.7227
Loop diuretic	89.20%	91.70%	0.6844

BMI—body mass index; SBP—systolic blood pressure; DBP—diastolic blood pressure; NYHA—New York Heart Association; HF—heart failure; CAD—coronary artery disease; DCM—dilated cardiomyopathy; ICD—implantable cardioverter-defibrillator; CRT-D—cardiac resynchronization therapy defibrillator; AF—atrial fibrillation; COPD—chronic obstructive pulmonary disease; WBC—white blood cells; RBC—red blood cells; HGB—hemoglobin; RDW—red blood cell distribution width; PLT—platelets; ESR—erythrocyte sedimentation rate; CRP—C-reactive protein; Na^+^—sodium; K^+^—potassium; NT-proBNP—N-terminal prohormone of brain natriuretic peptide; TSH—thyrotropin; Peak VO_2_—peak oxygen consumption; VE/VCO_2_ slope—minute ventilation/carbon dioxide production; PETCO_2_—partial pressure of end-tidal carbon dioxide; LVEF—left ventricle ejection fraction; ACE-I—angiotensin-converting enzyme inhibitors; ARB—angiotensin II receptor blocker; ARNI—angiotensin receptor neprilysin inhibitor; MRA—mineralocorticoid receptor antagonist.

## Data Availability

The data presented in this study are available on request from the corresponding author.
